# Regulators of *Salmonella*-host interaction identified by peripheral blood transcriptome profiling: roles of TGFB1 and TRP53 in intracellular *Salmonella* replication in pigs

**DOI:** 10.1186/s13567-018-0616-9

**Published:** 2018-12-12

**Authors:** Tinghua Huang, Xiali Huang, Bomei Shi, Fangfang Wang, Wenzhao Feng, Min Yao

**Affiliations:** College of Animal Science, Yangtze University, Jingzhou, 434025 Hubei China

## Abstract

Peripheral blood transcriptome is an important intermediate data source for investigating the mechanism of *Salmonella* invasion, proliferation, and transmission. We challenged 4-week old piglets with *Salmonella enterica* serovar Typhimurium LT2 and investigated the peripheral blood gene expression profile before treatment (d0) and at 2 and 7 days post-inoculation (dpi) using deep sequencing. Regulator pathways were first predicted in silico and validated by wet-lab experiments. In total, 1255, 765, and 853 genes were differentially expressed between 2 dpi/d0, 7 dpi/d0, and 7 dpi/2 dpi, respectively. Additionally, 1333 genes showed a time effect during the investigated *Salmonella* infection period. Clustering analysis showed that the differentially expressed genes fell into six distinct expression clusters. Pathway annotation of these gene clusters showed that the innate immune system was first significantly upregulated at 2 dpi and then attenuated at 7 dpi. Toll-like receptor cascades, MyD88 cascade, phagosome pathway, cytokine signaling pathway, and lysosome pathway showed a similar expression pattern. Interestingly, we found that the ribosome pathway was significantly inhibited at 2 and 7 dpi. Gene expression regulation network enrichment analysis identified several candidate factors controlling the expression clusters. Further in vitro study showed that TGFB1 can inhibit *Salmonella* replication whereas TRP53 can promote *Salmonella* replication in porcine peripheral blood mononuclear cells and murine macrophages. These results provide new insights into the molecular mechanism of *Salmonella*-host interactions and clues for the genetic improvement of *Salmonella* infection resistance in pigs.

**Electronic supplementary material:**

The online version of this article (10.1186/s13567-018-0616-9) contains supplementary material, which is available to authorized users.

## Introduction

*Salmonella enterica* is a Gram-negative intracellular bacterium with more than 2500 serotypes. Serovars such as Typhimurium and Choleraesuis that colonize both animals and humans are of public health importance [1]. In pigs, *Salmonella enterica* is the cause of asymptomatic carriage status to systemic febrile infection-related death varying with the ages of the animals [2]. *Salmonella* carrier pigs can transmit bacteria to other animals raised in the same pen or may contaminate the pig carcass in the slaughterhouse, thus posing a significant threat to the swine industry [3]. Establishment of carrier status is determined by the pathogenicity of serovars causing the infection as well as the genetic predisposition of the infected individual [4]. Several important *Salmonella* resistance genes have been identified in animals including SLC11A1 [5], TLR4 [6], LBP, and CD14 [7], NADPH oxidase and NOS2 [8], TNF and IFNG [9, 10], IL12 [11], TGFB1 [12], TRP53 [13], and more remaining to be discovered. The TGFB1 gene encodes a secreted ligand of the TGF-beta superfamily, binds various TGF-beta receptors that regulate gene expression, cell proliferation, differentiation and growth, and can modulate expression and activation of other growth factors including IFNG and TNF [9, 10]. The TRP53 gene encodes tumor protein p53, which responds to diverse cellular stresses to regulate target genes that induce cell cycle arrest, apoptosis, senescence, DNA repair, or changes in metabolism [14, 15].

An alternative approach for identifying the key genes controlling *Salmonella* infection is to measure host responses to *Salmonella* [16–20], and screening the host mRNA responses [21] in immune tissues such as the lungs, Peyer’s patches, or lymph nodes using real-time PCR [22] or microarrays [23–25]. Evidence from systemic typhoid infections indicate that some of the ingested bacteria invade epithelial and M cells, and are engulfed by resident macrophages in the submucosa [26, 27]. Casp1-mediated macrophage death and escape of bacteria from the intracellular environment allows further systemic dissemination of *Salmonella* to the bloodstream either by entering the blood directly or by passing through lymphatic vessels and mesenteric lymph nodes [28]. In the blood stream, bacteria transport to distant sites to reach an intracellular location in the liver, spleen, or bone marrow [28, 29]. In these processes, the blood stream is an important inter-medium window for *Salmonella* invasion, transportation, and proliferation, to finally exert its virulence. To date, there have been many reports on the responses of the transcriptome in porcine whole blood or peripheral blood derived cell populations to infection with bacteria or viruses [25, 30–36], immune stimulants [37], or following vaccination [38]. Several reports have demonstrated the genetic control of various immune cell parameters or immunological traits [39–44], as well as loci associated with susceptibility to salmonellosis [45, 46].

The peripheral blood transcriptome may be reflected in the gene expression response to *Salmonella* inoculation from early invasion to late carrier establishing stages. Therefore, studies have been performed on 40 piglets to determine the spectrum of transcriptional regulation by *Salmonella enterica* serovar Typhimurium inoculation [25, 47]. Two groups of pigs with either low shedding (LS) or persistent shedding (PS) phenotypes were identified. Global transcriptional changes in response to *Salmonella* inoculation were investigated using Affymetrix Genechip^®^ analysis of peripheral blood RNA at d0 and 2 dpi. *Salmonella* inoculation triggered substantial gene expression changes in the pigs and 217 genes were differentially expressed between LS and PS pigs. Analysis of the differential gene expression profiles revealed distinct regulatory pathways mediated by IFN-γ, TNF, and NF-κB. These data provided useful information on the early immune response to *Salmonella* infection in pigs, however, the later stages remain unknown.

In the current study, we challenged piglets with *Salmonella enterica* serovar Typhimurium LT2, a genome sequencing completed *Salmonella* serovars, and investigated the gene expression profile in peripheral blood at d0, 2 dpi, and 7 dpi using deep sequencing technology. We identified two novel regulatory genes that downregulate host genes. Further studies indicated that these two genes play roles in regulating intracellular *Salmonella* replication in porcine PBMCs and murine macrophage cells. This study provides new data on peripheral blood transcriptomic responses to *Salmonella enterica* serovar Typhimurium challenge in piglets and reveals the regulatory pathways and key genes controlling these gene expression responses. These genes can be targeted for further exploration on the development of carrier status. This knowledge can also be used for rational manipulation of genetics, pharmaceuticals, and nutrition or husbandry methods to decrease *Salmonella* colonization, shedding, and spread.

## Materials and methods

### Animal challenge, sample collection, and processing

Peripheral blood samples were collected from 30 piglets (bought from a commercial swine farm) challenged with *Salmonella enterica* serovar Typhimurium LT2 using a previously described method [47]. In brief, piglets of the Duroc × Landrace × Yorkshire crossbreed were used. Piglets were raised in fully enclosed climate-controlled isolation facilities. The pig fecal matter tested negative for *Salmonella* three times before intranasal challenge with 10^9^ colony forming units (cfu) of *Salmonella enterica* serovar Typhimurium LT2 at 4 weeks of age. Blood samples were collected from each animal at d0 or at 2 and 7 dpi (10 animals per group). Peripheral whole blood (approximately 2.3 mL) was collected from the jugular vein into PAXgene Blood RNA tubes and processed according to the instructions provided by Qiagen. Total RNA for all animals was prepared from 4.5 to 9.0 mL solutions from the PAXgene Blood RNA tubes using the PAXgene Blood RNA kit (Qiagen, Cat. no. 762164). The quantity and quality of RNA samples were determined using the Agilent 2100 Bioanalyzer (Agilent Technologies, Santa Clara, CA, USA) and Nanodrop 2000 (Thermo Scientific, Wilmington, DE, USA). RNA samples with the RIN number lower than 7 or yield less than 3 μg were identified as low quality and excluded from the experiment. Four samples from each group (d0 or 2 and 7 dpi) were selected for transcriptome analysis.

### Deep sequencing and statistical analysis

Sequencing libraries were prepared using the Illumina Truseq RNA sample preparation kit according to the manufacturer’s protocol (Illumina Inc., USA). Approximately 10 μg of total RNA from each sample was used for library construction and RNA sequencing. Sequencing was conducted on an Illumina HiSeq 2000 (Illumina Inc., USA) by single-read sequencing (read length 50 bp). The images generated by the sequencer were converted into raw reads by base calling. Data filtering was conducted to obtain clean reads and to remove low quality reads present in the raw reads. The procedure includes the following steps: 1) Removal of reads in which unknown bases (N) are presented; 2) removal of low quality reads, i.e., if the reads have low quality bases (bases with quality value ≤ 5). Bowtie1 [48] was used to map clean reads to the reference genes set, which was extracted from the NCBI reference sequence database [49]. The calculated reads count per gene per length were used for comparing the difference in gene expression among samples. The limma R package [50] was used for the calculation and the criteria for differentially expressed genes were controlled as FDR (false discovery rate) ≤ 0.05 and fold change ≥ 1.5 or ≤ 0.67. All the data discussed in this study have been deposited to NCBI GEO database [51] under accession number GSE118150. The fixed effects for time along with random pig effects were fit to the expression data for each gene using SAS PROC MIXED and the p-values for each test were converted to FDR and controlled at ≤ 0.05 as well [52].

### Real-time PCR, ELISA, and functional annotation of differentially expressed genes

Pig peripheral blood was collected into citrate tubes (6 mL) from 20 animals and randomly divided into five groups. One group was used as a control and other groups were used in two separate experiments: (1) treated with TGF-β1 recombinant protein (240-B-010, R&D Systems, Inc, MN, USA), or it’s inhibitor (Pifithrin-α hydrobromide, R&D Systems); (2) treated with p53 recombinant protein (SP-454-020, R&D Systems), or it’s inhibitor (SB-431542, R&D Systems). The samples were incubated for 4 h at 37 °C with 5% CO_2_ under conditions reported previously for in vitro stimulation of whole blood [53]. RNA samples extracted from the peripheral blood of 20 animals with different treatment were used for real-time PCR. Reverse transcription was performed using SuperScript II Reverse Transcriptase and Oligo(dT) primers according to the manufacturer’s instructions (Invitrogen, Carlsbad, CA, USA). Real-time PCR was performed using a standard SYBR Green PCR kit (Applied Biosystems, Carlsbad, CA, USA) and a BIO-RAD iQ5 Real-Time PCR Detection System. All reactions were run in duplicate. The data were initially normalized using the averaged expression level of Beta-actin and GAPDH. The serum TGF-β1 and p53 protein levels were quantified using the DuoSet^*®*^ IC ELISA kit and Quantikine^®^ ELISA, respectively, according to the manufacturer’s instructions (R&D Systems). The most current porcine gene annotation was used to assign the transcripts to mouse RefSeq according to the dual best match method. Cluster and Treeview softwares were used to cluster the data [54]. The open-access bioinformatics tool, InnateDB [55], was used to identify significantly regulated pathways between different time points and shedding phenotypes. The significantly enriched gene expression regulators of these differentially expressed gene lists were identified by Gene Expression Regulator Enrichment Analysis (GEREA) developed in-house (Additional file 1).

### Intracellular *Salmonella* replication assays

*Salmonella* intracellular replication assays were carried out using a previously described protocol [56, 57]. RAW 264.7 (ATCC^®^ TIB-71™) cells were cultured in DMEM, supplemented with 10% FBS and 1% GlutaMAX (GIBCO) at 37 °C under 5% CO2. TGF-β1 and p53 recombinant proteins and inhibitors were incubated with the cells for 24 h prior to bacterial addition. Bacterial inoculation was carried out by adding 5 μL of *Salmonella* culture, centrifugation, followed by 2 h of incubation at 37 °C in a 5% CO_2_ atmosphere. The monolayers in microtiter cell plates were washed with PBS and then incubated for another 2 h in fresh media containing 100 μg/mL of gentamicin. This treatment kills the extracellular bacteria but does not affect the viability of intracellular organisms [57]. Monolayers were washed three times with PBS, and 0.2 mL of 1% Triton X-100 solution was added. This was followed by a 5-min incubation period to release the intracellular bacteria. L-broth was added, appropriate dilutions were spread onto L-agar plates, and bacterial counts were determined. The experiment was repeated three times.

## Results

### Gene expression changes in peripheral blood following challenge with *Salmonella enterica* serovar Typhimurium

**Figure 1:**
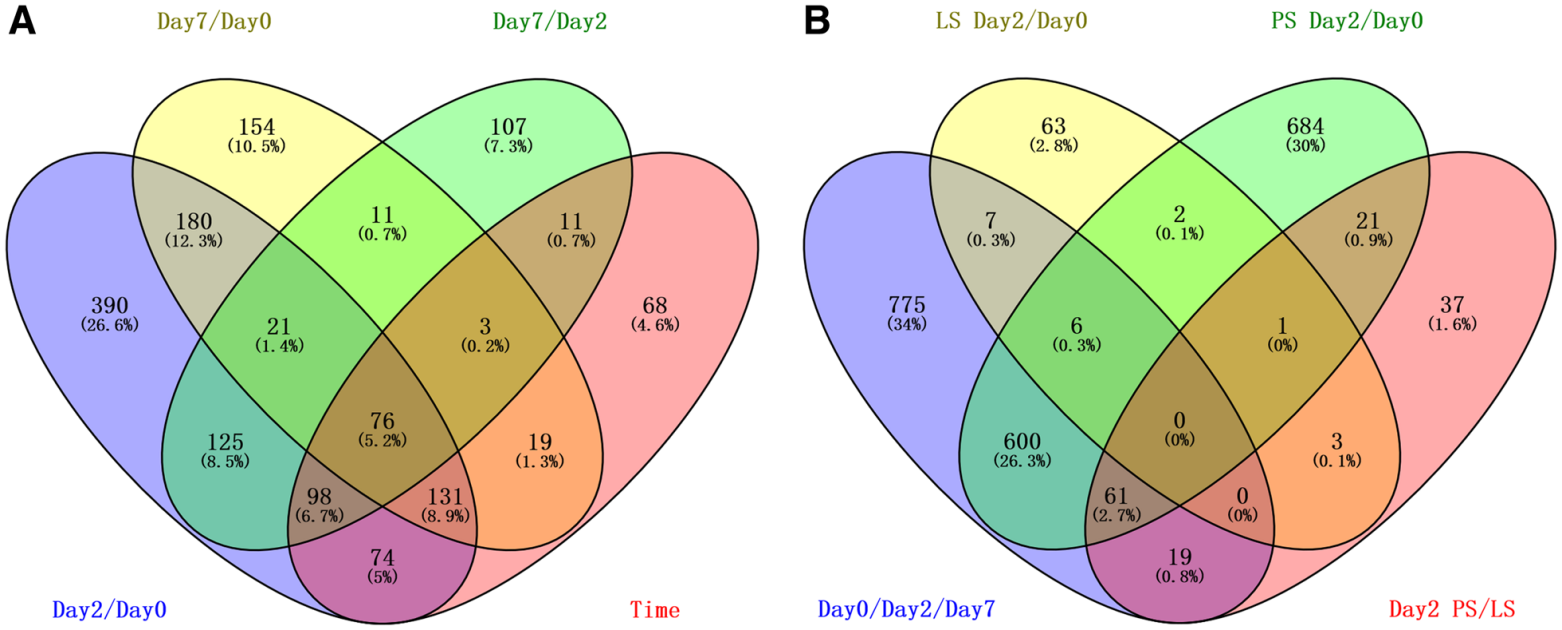
**Numbers of differentially expressed genes. A** Summary of differentially expressed transcripts responding to *Salmonella* inoculation (2 dpi/d0, 7 dpi/d0, 7 dpi/2 dpi), or expression showing time effect. **B** Comparison of differentially expressed gene counts in LS/PS animals as reported by Huang et al. [25]. These genes were identified using the linear mixed model, and the false discovery rate (FDR) was controlled at less than 0.05, with the fold change between time points required to be higher than 1.5 or less than 0.67.

Our previous investigations have demonstrated that the peak of both clinical symptoms (fever, diarrhea, decreased appetite) as well as *Salmonella* shedding occurs at 2 dpi and is attenuated at 7 dpi [25, 47]. Thus, the peripheral blood RNA expression data for d0, 2 dpi, and 7 dpi were profiled and the resulting data were analyzed as described in the Methods section. The complete data on gene expression are shown in Additional file 2. Direct comparison between different time points revealed 1095 transcripts that were differentially expressed between 2 dpi and d0 and 595 transcripts differentially expressed between 7 dpi and d0 after *Salmonella enterica* serovar Typhimurium inoculation. We also observed 452 transcripts differentially expressed between 7 dpi and 2 dpi. In total, 480 transcripts were statistically significant (FDR < 0.05) for tests of time effects. The numbers of differentially expressed transcripts (NCBI Reference Sequence) for each comparison, and the numbers of transcripts overlapping among comparisons (comparing 2 dpi/d0, 7 dpi/d0, or 7 dpi/2 dpi), are shown in Figure 1A, full lists are available in Additional file 3. As expected, we observed significant variation in the RNA levels for 53 genes responding to IFN-γ stimulation [58], including Casp1, CASP4, CD14, CEBPB, IRF3, SLC11A1, SPI1, and TLR4, along with 55 genes responding to TNF stimulation [58], including MAPK14, TLR4, CASP1, CD47, CEBPB, CSF2RB, GADD45B, and others. The mRNA levels for innate inflammatory marker genes such as SLC11A1 and TLR4 were strongly changed following *Salmonella* inoculation. Comparison with differentially expressed gene counts in peripheral blood after *Salmonella* inoculation in pigs as reported by Huang et al. [25] revealed a total of 13 genes in common with LS animals, 666 genes in common with PS animals, and 80 genes in common upon PS/LS comparison (Figure 1B). Interestingly, we found a total of 775 genes that were differentially expressed in piglets challenged with *Salmonella enterica* serovar Typhimurium LT2, which were unique to LS and PS animals.

### Distinctive mRNA expression patterns during *Salmonella enterica* serovar Typhimurium challenge in peripheral blood

**Figure 2:**
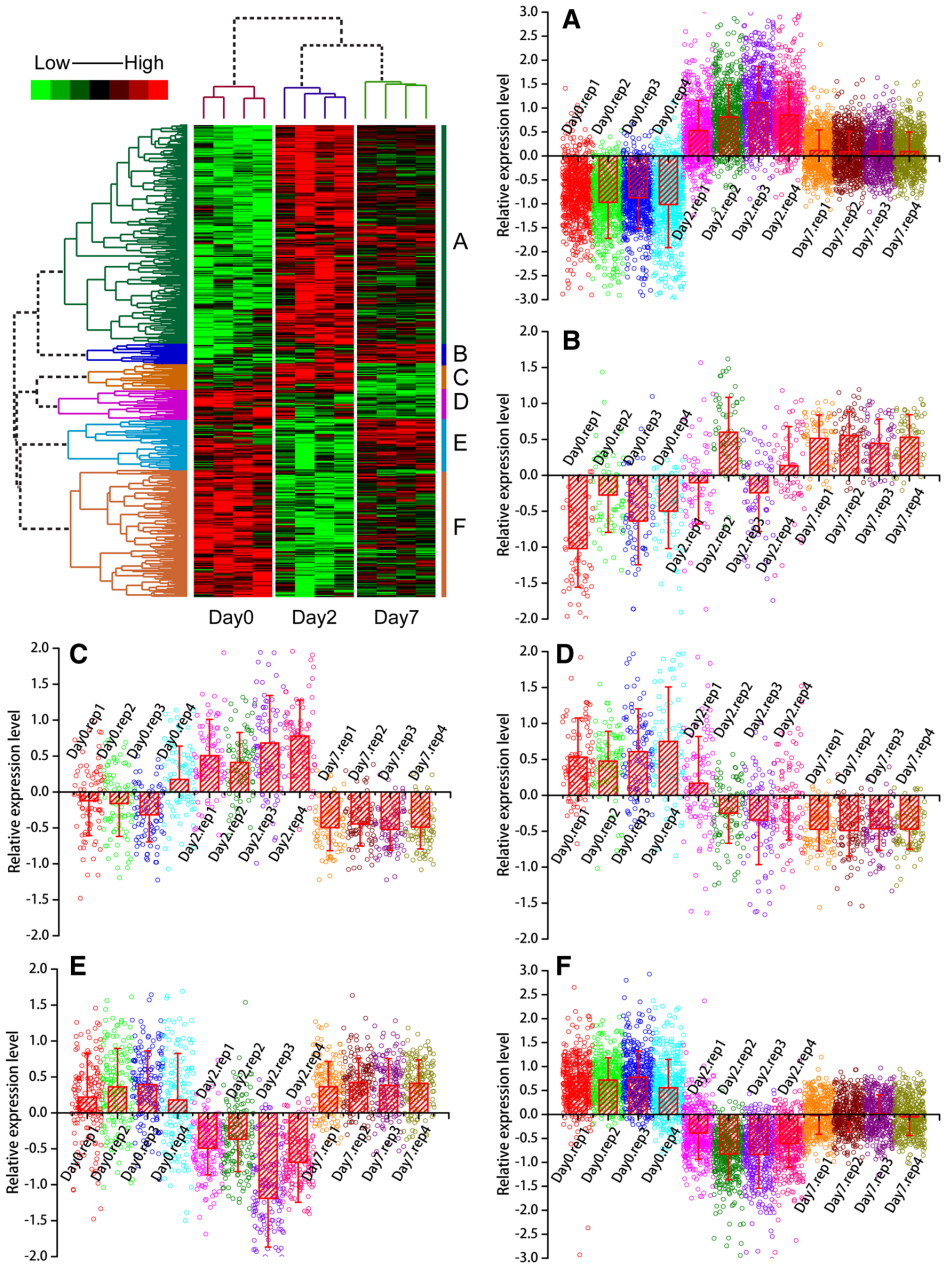
**Hierarchical cluster analysis of differentially expressed genes.** We performed data adjustment (median center and normalization) in the cluster analysis. The color codes red, white, black, and dark green represent high, average, low, and absent expression, respectively. A detailed view of gene expression levels in clustering patterns is shown in plot areas from **A**–**F**.

To visually illustrate the expression type of mRNAs being expressed during *Salmonella enterica* serovar Typhimurium challenge in peripheral blood, hierarchical cluster analysis was performed for the genes differentially expressed between 2 dpi/d0, 7 dpi/d0, 7 dpi/2 dpi, and genes showing time effects. The results showed that the mRNA expression patterns fall into six typical categories: (A) activated at 2 dpi, expression level increased between 2 dpi and d0 and then moderately expressed at 7 dpi; (B) activated at 7 dpi, low expressed at d0, expression level increased at 7 dpi; (C) activated at 2 dpi and dropped at 7 dpi, expression level increased between 2 dpi and d0 and then decreased at 7 dpi; (D) down-regulated at both 2 dpi and 7 dpi, expression levels lower at both 2 dpi and 7 dpi compared with d0; (E) downregulated at 2 dpi and upregulated at 7 dpi, expression levels are significantly lower at 2 dpi and higher at 7 dpi compared with d0; (F) downregulated at 2 dpi and attenuated at 7 dpi, the expression levels are significantly lower at 2 dpi compared with d0 with moderate expression at 7 dpi. The expression patterns described above are clearly reflected by the formation of several large clusters in the tree map of the clustering results (Figure 2).

### Functional annotation of genes showing similar expression pattern

Pathway annotation of the six gene clusters showed that the innate immune system, Toll-like receptors cascades, MyD88 cascade, phagosome and lysosome pathway, cytokine signaling pathway, and lysosome pathway were over-represented in Cluster A. In total, 55 immune genes were presented in Cluster A wherein 27 were related to the innate immune system and 23 were related to the adaptive immune system. We also found 16 phagosome genes and 15 lysosome genes in Cluster A. In total, 8.6% genes in Toll-like receptors cascades are presented in Cluster A and 10 of these are involved in the TLR4 cascade. The cytokine signaling system was also significantly over-represented in Cluster A. In Cluster B, there were no significantly over-represented pathways. In Cluster C, metabolism and disease pathways were significantly over-represented. In Cluster D, cell cycle-related pathways were over-represented, 8 genes were related to the mitotic pathway and 6 genes were related to the G1 − G1/S phases. In Cluster E, genes related to expression were over-represented. More specifically, 9 genes were related to translation pathways and 8 genes were from the eukaryotic translation initiation, elongation and termination pathway. Cluster F was also related to expression. In this cluster, 15 genes were involved in the translation pathway, 14 genes were related to ribosome; more specifically, 13 genes were related to translation elongation, 13 genes were related to translation initiation, and 12 genes were related to translation termination. The significantly over-represented pathways in cluster A, C, D, E, and F are listed in Additional file 4.

**Figure 3:**
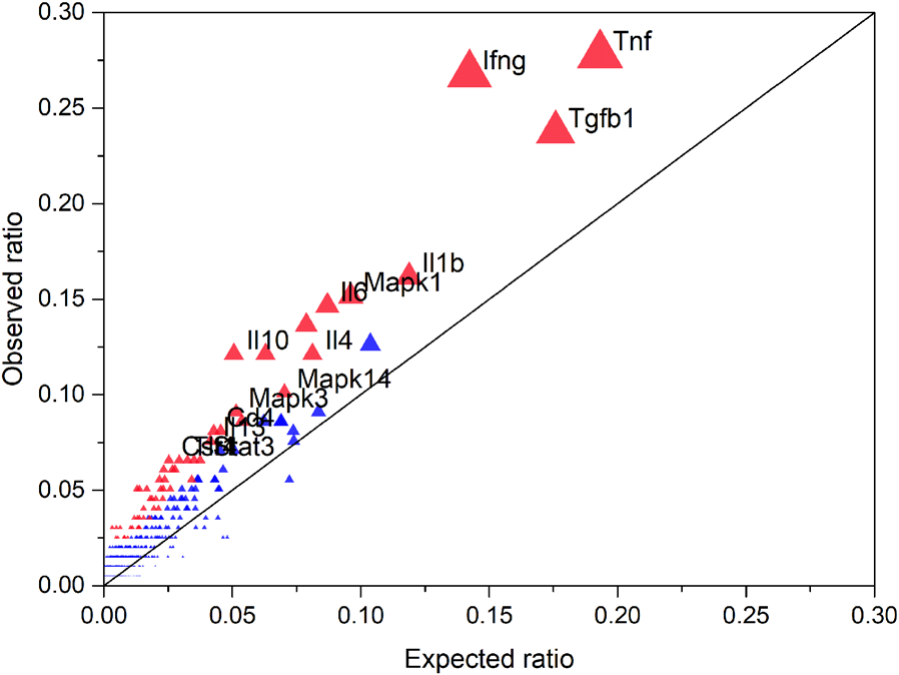
**Gene expression regulator enrichment analysis using the genes in Cluster A.** The significantly over-represented regulators were plotted based on the ratio of targets to the total number of transcripts in the differentially expressed gene list (observed) and all genes presented in the transcriptome (expected). Red color indicates that the regulator is statistically significant whereas blue indicates no significance. The size of the triangle indicates the number of targets for the regulator.

Using regulator-target relationships known from PubMed literature, we used Gene Expression Regulator Enrichment Analysis (GEREA) to identify key “regulators” whose connections to specific sets of differentially expressed target genes were over-represented (FDR < 0.05, Figure 3 and Table 1). Such relationships include direct interactions as well as more indirect relationships inferred from published sources. Very few common regulators of target genes were significantly over-represented in the lists of Cluster B and C. However, in Cluster A, many regulator-target relationships were statistically significant, including cytokines such as TNF, IFN-γ, and immune-inflammatory-related transcription factors such as MAPK14 and STAT3, which were linked to the largest number of differentially expressed genes, indicating their important roles in the regulation of gene expression responses to *Salmonella enterica* serovar Typhimurium infection. Four interleukins (IL1-β, IL6, IL4, and IL13) were also significantly enriched. These interleukins have extensive effects on T cell activation, B cell proliferation, natural killer cell activation, and antibody production, and also have broad interactions with other immune and inflammatory modulators such as IFN-γ and NF-κB. Several other genes with altered expression patterns following *Salmonella* inoculation, including IRFs and STATs, as well as SPI1, and TLR4, showed significantly enriched regulator-target sets. The regulator of TGFB1, whose protein product acts as transforming growth factor, was also significantly enriched with a high number of target genes. The full list of regulators is available in Additional file 5.

**Table 1:** Significantly over-represented regulators in clusters D, E, and F

Cluster name	Pathway regulator	Gene count in pathway	Gene count in cluster	P0^a^	P1^b^	FDR^c^
Cluster D	Egf	459	8	0.084	0.364	0.000
Cluster D	Mapk1	528	6	0.096	0.273	0.013
Cluster D	Ppara	255	5	0.046	0.227	0.003
Cluster D	Mapk3	283	5	0.052	0.227	0.004
Cluster D	Lep	295	5	0.054	0.227	0.005
Cluster D	Mapk14	386	5	0.070	0.227	0.014
Cluster D	Trp53	405	5	0.074	0.227	0.016
Cluster D	Il4	446	5	0.081	0.227	0.025
Cluster D	Il6	478	5	0.087	0.227	0.031
Cluster E	Tnf	1061	17	0.193	0.370	0.003
Cluster E	Tgfb1	966	15	0.176	0.326	0.007
Cluster E	Fgf2	397	8	0.072	0.174	0.015
Cluster E	Il4	446	8	0.081	0.174	0.024
Cluster E	Creb1	247	7	0.045	0.152	0.005
Cluster E	Bdnf	123	6	0.022	0.130	0.001
Cluster E	Ifnb1	168	6	0.031	0.130	0.003
Cluster E	Cd8a	159	6	0.029	0.130	0.003
Cluster E	Hgf	237	6	0.043	0.130	0.013
Cluster E	Il2	245	6	0.045	0.130	0.015
Cluster E	Notch1	255	6	0.046	0.130	0.016
Cluster E	Csf2	249	6	0.045	0.130	0.019
Cluster E	Sirt1	89	5	0.016	0.109	0.001
Cluster E	Pten	102	5	0.019	0.109	0.002
Cluster E	Csf3	127	5	0.023	0.109	0.005
Cluster E	Src	188	5	0.034	0.109	0.017
Cluster E	Edn1	188	5	0.034	0.109	0.021
Cluster E	Egfr	218	5	0.040	0.109	0.034
Cluster E	Bmp4	197	5	0.036	0.109	0.035
Cluster E	Vegfa	250	5	0.046	0.109	0.043
Cluster F	Trp53	405	14	0.074	0.128	0.017
Cluster F	Mapk14	386	13	0.070	0.119	0.027
Cluster F	Cd8a	159	7	0.029	0.064	0.047
Cluster F	Gata1	88	5	0.016	0.046	0.031

^a^An expected ratio in the background.

^b^An observed ratio in the list of differentially expressed gene.

^c^Statistical analysis was performed based on cumulative hypergeometric distribution and corrected by Benjamini and Hochberg method.

In Cluster D, nine regulator-target relationships were statistically significant, including EGF, which is implicated in cell proliferation and differentiation, MAPK1 that is involved in a wide variety of cellular processes such as proliferation, differentiation, transcription regulation, and development, and TRP53 that responds to diverse cellular stresses to regulate target genes that induce cell cycle arrest and apoptosis (Table 1). In Cluster E, a total of 20 regulator-target relationships were statistically significant including TNF, TGFB1, FGF2, IL4, and CREB1 (Table 1). Some of these regulators such as TNF and TGFB1, were also statistically significant in Cluster A. As the regulation relationships from the regulator to the targets include both direct interactions as well as more indirect relationships, *Salmonella* may target downstream hubs of these regulators and inhibit a sub-network. In Cluster F, TRP53, MAPK14, CD8A, and GATA1 were statistically significant, whereas TRP53 regulated the largest number of target genes (Table 1). TRP53 encodes the tumor suppressor protein p53, which responds to diverse cellular stresses to regulate target genes that induce cell cycle arrest and apoptosis. Interestingly, the cell cycle-related pathways were downregulated at both 2 dpi and 7 dpi (over-represented in Cluster D), suggesting that TRP53 may be an important target of *Salmonella*. The relevant functions of other over-represented regulators and subnetworks of regulated targets are less defined.

### TNF-α, IFN-γ, TGFB1, and TRP53 profiles in *Salmonella* challenged pigs

**Figure 4:**
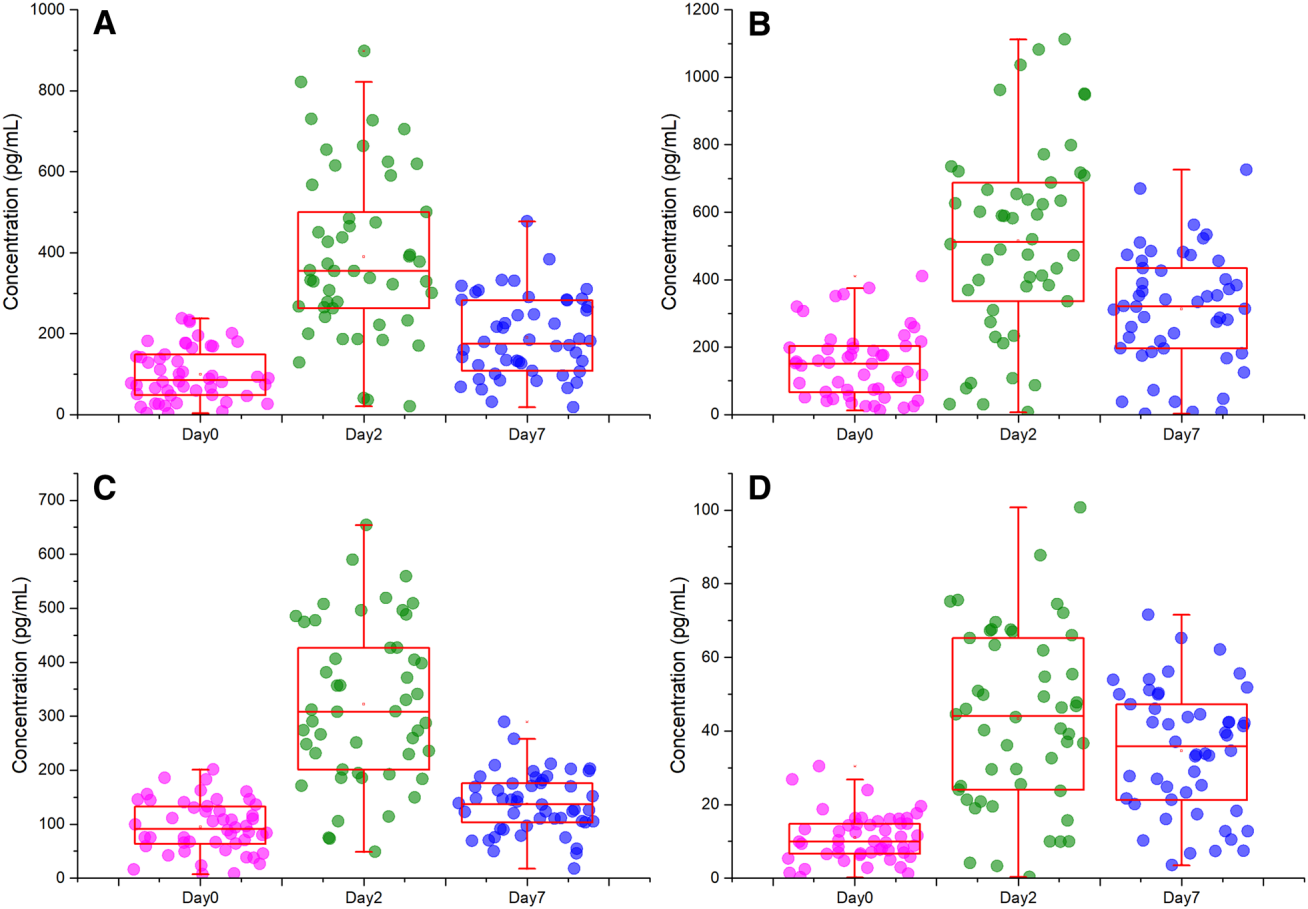
**Peripheral blood concentrations in** ***Salmonella*-challenged pigs.** Serum protein levels were measured prior to challenge and at 2 and 7 dpi using ELISA. Results are presented as box plots plus scatter plots for each sample. Plots **A**–**D** show the results of TNF-α, IFN-γ, TGFB1, and TRP53 respectively.

We measured the serum concentrations of top enriched gene expression regulators in Cluster A (TNF-α, IFN-γ, TGF-β1), and in Cluster F (p53) as biomarkers of inflammation and gene expression regulation to identify the potential response differences introduced by *Salmonella* challenge (Figure 4). Prior to challenge, serum samples from the 30 pigs presented similar concentrations of these proteins when compared to control pigs (d0). However, at 2 dpi, distinct differences emerged in pigs compared to those at d0. The serum collected at 2 dpi from pigs showed elevated TNF-α, IFN-γ, TGF-β1, and p53 concentrations compared to those at d0 (*p* ≤ 0.05). Pigs at 7 dpi showed decreased concentrations of TNF-α and TGF-β1 compared with those at 2 dpi (*p* ≤ 0.05), but still higher than those at d0. IFN-γ concentrations were lower at 7 dpi than at 2 dpi, but were still significantly higher than those at d0 (*p* ≤ 0.05). The concentrations of TRP53 remained elevated at 7 dpi (not significant compared to 2 dpi) and were four folder higher than those at d0.

### Expression of target genes in response to in vitro treatment with TGF-β1 and p53

To determine if the in vivo expression patterns of target genes of two of the top enriched gene expression regulators, TGFB1 and TRP53, could be regulated by recombinant proteins as well as protein inhibitor treatment, we used real-time PCR after in vitro treatment with two different doses of recombinant proteins (1 ng/mL and 10 ng/mL) and TGFB1 and TRP53 inhibitors (0.1 µmol/mL and 0.5 µmol/mL). Samples were collected at 4 h post-stimulation and subjected to real-time PCR using the primers listed in Additional file 6. In total, 28 randomly selected target genes (14 for each) were selected for measurement. Thirteen out of 14 TGFB1 targets showed upregulation upon treatment with 1 ng/mL recombinant protein. All 14 TGFB1 targets were upregulated when treated with 10 ng/mL recombinant protein. When TGFB1 inhibitor was added, the target genes showed no response at all. Four out of 14 TRP53 targets were downregulated when treated with 1 ng/mL recombinant protein. Thirteen out of 14 TRP53 targets showed downregulation when treated with 10 ng/mL recombinant protein. When TRP53 inhibitor was added, the target genes showed no response at all. The raw data and statistical results for real-time PCR are shown in Additional file 7.

**Figure 5:**
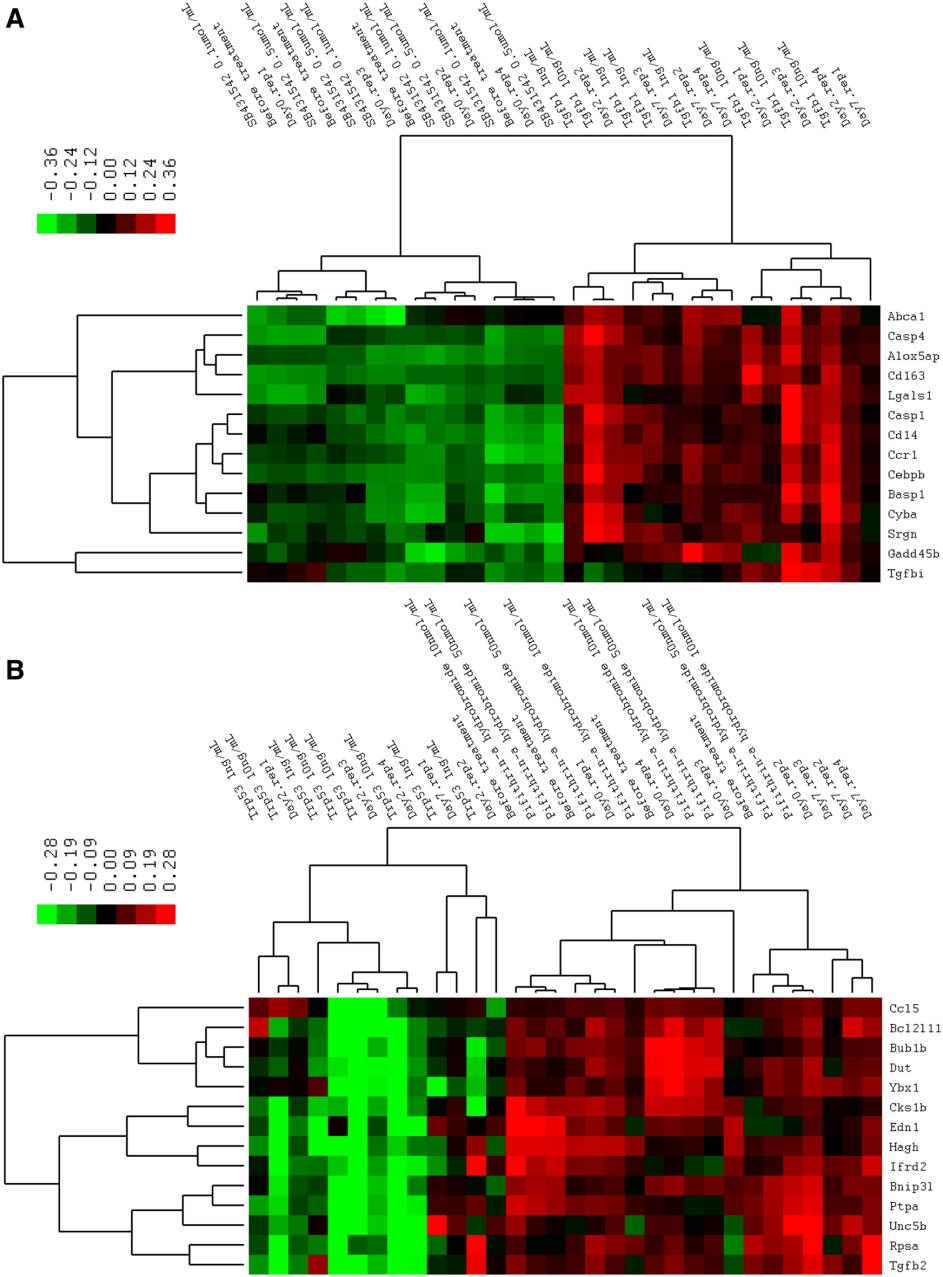
**Hierarchical clustering of gene expression data from peripheral blood measured by real-time PCR.** Peripheral blood samples were treated in vitro with two different doses of recombinant TGFB1 and p53 protein (1 ng/mL and 10 ng/mL, panel A) and TGFB1 and TRP53 inhibitors (0.1 µmol/mL and 0.5 µmol/mL, panel B). Color codes of yellow, black, and blue represent high, average, and low expression levels, respectively, across the treatments shown.

To determining whether the treatment response pattern of combined target genes was similar or different to the patterns detected upon in vivo *Salmonella* challenge, hierarchical cluster analysis of the average mRNA levels of 28 target genes was performed. The results indicated that the expression patterns of samples treated with recombinant TGF-β1 protein were clustered with the 2 dpi *Salmonella* challenge group. The before treatment group and TGFB1 inhibitor treatment group were mixed up with the 0 dpi and 7 dpi *Salmonella* challenged groups. No dose effect of recombinant TGF-β1 protein and its inhibitor was observed as the 1 ng/mL TGF-β1 group was clustered together with the 10 mg/mL TGF-β1 group and the 0.1 µmol/mL inhibitor group was clustered together with the 0.5 µmol/mL inhibitor group in the tree map. The most similar pattern seen upon recombinant p53 protein treatment was in the 2 dpi and 7 dpi *Salmonella* challenge group. The recombinant p53 protein treatment group was most similar to the 2 dpi *Salmonella* challenged group, whereas the TRP53 inhibitor treatment group was clustered together with the 7 dpi *Salmonella* challenged group as well as the before treatment and challenge group. The cluster result of TGFB1 is shown in Figure 5A and that for TRP53 is showed in Figure 5B.

### TGFB1 and TRP53 regulate intracellular *Salmonella* growth

**Figure 6:**
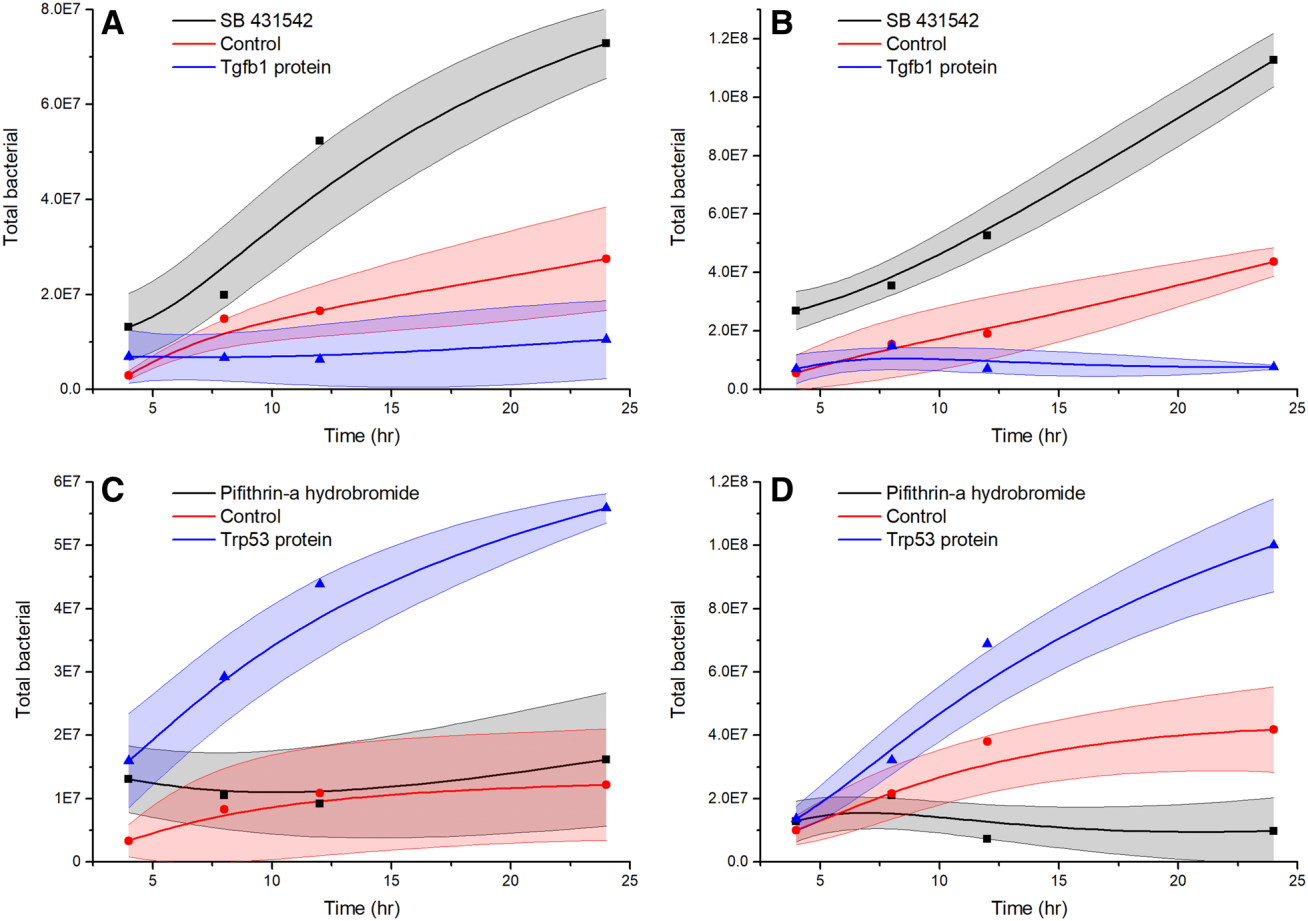
**Intracellular growth of** ***Salmonella enterica*** **serovar Typhimurium strain LT2 in macrophages.** Macrophages were first treated with recombinant protein or protein inhibitor, followed by infection with *Salmonella* bacteria, incubation for 2 h, treatment with gentamicin (100 µg/mL) for 2 h to kill the extracellular bacteria, and immediate lysis or incubation for an additional 8, 12, or 24 h. Line graphs show the intracellular *Salmonella* counts after the macrophages were treated with *Salmonella* bacteria. The filled areas represent the standard deviation of three duplicate samples. **A** PBMCs treated with recombinant TGFB1 or TGFB1 inhibitor; **B** murine macrophages (RAW 264.7) treated with recombinant TGFB1 or TGFB1 inhibitor; **C** PBMCs treated with recombinant TRP53 or TRP53 inhibitor; **D** murine macrophages (RAW 264.7) treated with recombinant TRP53 or TRP53 inhibitor.

PBMCs were first treated with recombinant TGFB1, recombinant TRP53, or their inhibitors and were then treated with *Salmonella* to examine how two genes would affect the intracellular *Salmonella* growth (Additional file 8). The results showed that after treatment with recombinant TGFB1, the intracellular bacteria in PBMCs were significantly lower at 12 and 24 h post-treatment compared to the results obtained in the control experiment (*p* < 0.05, Figure 6A). The intracellular bacterial growth in PBMCs was continually increased over time in cells treated with the TGFB1 inhibitor, and was significantly higher at 12 and 24 h post-treatment than in the control experiment. In murine macrophages (raw 264.7), intracellular bacterial growth is similar to that within PBMCs as recombinant TGFB1 treatment decreased the intracellular bacterial growth whereas the inhibitor increased bacterial growth. Treatment of PBMCs with TRP53 inhibitor had no effect on intracellular bacterial growth (there was no difference between TRP53 inhibitor treatment and the control experiment). However, after treatment with the recombinant p53 protein, intracellular bacterial growth was significantly higher at 4, 12, and 24 h post-treatment compared with the TRP53 inhibitor treatment and the control experiment (*p* < 0.05, Figure 6C). In murine macrophages, the effect of recombinant TRP53 and TRP53 inhibitor treatment is clear, wherein treatment with recombinant p53 protein increased the intracellular bacterial growth at 12 and 24 h post-treatment and treatment with the TRP53 inhibitor decreased the intracellular bacterial growth at 12 and 24 h post-treatment (*p* < 0.05, Figure 6D).

## Discussion

### Peripheral blood transcriptome activation associated with *Salmonella* Typhimurium challenge

The genes responding to *Salmonella* challenge at 2 and 7 dpi indicated that the innate immune genes constituted the majority of over-represented responses and this was confirmed by InnateDB and GEREA analysis. InnateDB over-representation analysis identified that the TLR4 and IFN-γ systems are major inducers of transcriptomic responses in the peripheral blood of pigs. Significant upregulation of mRNA for TLR4 and elevation of over 9.2% genes in the TLR signaling pathway at 2 dpi indicated that this pathway was extensively activated at an early stage in response to *Salmonella*. The activated downstream cascades of the cytokine signaling pathway, Myd88 cascade, IFN-γ cascade, phagosomes, and lysosomes together with the Toll-like receptor signaling pathway itself cover most of the over-represented pathways, further indicating primary roles for TLR4 and IFN-γ systems in this response. GEREA analysis confirmed the importance of TLR4 and IFN-γ, and also identified other important regulators such as TNF (highest numbers of differentially expressed gene connected to this regulator), MAPKs (Mapk1, Mapk3, and Mapk14), and interleukins IL1β, IL6, IL4, and IL13.

Most importantly, the serum protein level of IFN-γ and TNF, the top ranked regulator for the differentially expressed genes in Cluster A (cluster with largest numbers of genes), was increased after *Salmonella* inoculation. IFN-γ, produced predominantly by T lymphocytes and natural killer cells [59], is believed to prime macrophages to respond more vigorously to LPS. IFN-γ signaling also induces expression of several TLR signaling components including TLR4, which was differentially expressed in pig peripheral blood in response to *Salmonella* inoculation (13 fold for 2 dpi/d0, sixfold for 7 dpi/d0, 0.5 fold for 7 dpi/2 dpi), and has been proposed to remodel the initial NF-κB signaling [59]. TNF is a pleiotropic pro-inflammatory cytokine produced mainly by macrophages but also by activated NK cells and Th1 lymphocytes [9]. TNF plays a key role in the host defense against pathogens through several mechanisms including activation of neutrophils and platelets, enhancement of the killing activity of macrophages and NK cells, and activation of the immune system. It is encoded by the *TNF* gene and exerts its effects through two types of receptors, the TNFRP55 (encoded by *TNFRSF1A*, TNF receptor superfamily 1A gene) and TNFRP75 (*TNFRSF1B*). The early phase of bacterial killing within the macrophages is associated with activation of the NADPH oxidase system and the TNF receptor, TNFRP55 is necessary for targeting NADPH phagocyte oxidase-harboring vesicles to SCVs (*Salmonella* containing vacuoles) [60]. The upregulation of IFN-γ and TNF in serum and their roles in gene regulation indicate their important roles in *Salmonella* infection in pig.

### Genes with decreased expression in *Salmonella* Typhimurium challenge associated with ribosome function

A clearly intriguing result is the gene Clusters D, E, and F, which are mostly downregulated upon inoculation with *Salmonella* and were identified as differentially expressed in pig peripheral blood (Figure 2). Many of the over-represented terms in this set of genes are related to ribosomes and RNA translation. The ribosome translates genetic information into peptides through several steps including initiation, elongation, termination, and circulation [61]. Interestingly, the translation elongation, translation initiation, and translation termination were all significantly over-represented in Clusters E and F. The significantly enriched regulators that have the highest number of targets are TNF, TGFB1, TRP53, and MAKP14. The roles of TNF and MAKP14 in *Salmonella* infection are relatively clear whereas other regulators such TGFB1 and TRP53 are not. It would be quite interesting to explore the role of these regulators in *Salmonella* colonization, as the decreased target gene expression of these regulators indicates an important mechanism of *Salmonella* bacteria regulating the host gene expression in animals.

### TGFB1 and TRP53 regulate intracellular *Salmonella* replication in pig macrophages

The *Salmonella* intracellular replication assay indicated that TGFB1 can inhibit *Salmonella* replication and that TRP53 can promote *Salmonella* replication. TRP53 is a tumor suppressor gene known to balance cell survival and death in response to a variety of intrinsic and extrinsic stress signals [14, 15]. In response to *Salmonella* challenge, the TRP53 serum level was increased. Upregulation of TRP53 may lead to either cell-cycle arrest or apoptosis [62, 63]. Viral infections have also been shown to affect TRP53 activity by inducing type 1 IFN-mediated TRP53 mRNA upregulation [64]. On the other hand, TRP53 activation enhances IFN signaling [65]. During *Salmonella* infection, we observed both an increase in IFN secretion and enhanced TRP53 expression as well as TNF production. TRP53 can direct E2f2-mediated growth arrest involving the target gene GADD45 [66]. GADD45 was upregulated in *Salmonella* challenged pigs (6.8 fold for 2 dpi/d0, 5.4 fold for 7 dpi/d0), which further supports the hypothesis that after *Salmonella* inoculation, cells are maintained in a cell-cycle arrested state. It has been documented that other bacterial pathogens have elaborate sophisticated mechanisms to block cell-cycle progression favoring conditions for colonization and dissemination [13, 67]. Here, we showed that TRP53 expression is affected by *Salmonella* challenge in peripheral blood and that TRP53 treatment can promote intracellular *Salmonella* growth in macrophages. We speculate that its impact on *Salmonella* infection can be explained by favoring a cell-cycle arrested state allowing the bacteria to proliferate more successfully.

The effect of in vivo administration of recombinant TGF-β1 on the pathogenic mechanisms involved in experimental *Salmonella* Typhimurium infection in mice has been reported [12]. The protective response elicited by macrophages was induced by recombinant TGF-β1 in 2 days after experimental infection, as demonstrated by increased NO production [12]. The mice that received TGF-β1 survived after infection and the number of bacteria recovered in the spleens and livers of TGF-β1-treated mice after infection was significantly smaller than that found in the same organs after phosphate-buffered saline (PBS) inoculation [12]. In the present study, TGFB1 serum levels were increased in response to *Salmonella* challenge in pigs. Most importantly, macrophages treated with recombinant TGF-β1 protein showed increased resistance to intracellular *Salmonella* growth. Upregulation of TGFB1 regulates cell proliferation, differentiation, and growth, and can modulate the expression and activation of other growth factors including IFN-γ and TNF [68, 69]. We speculate that its effect on resistance to *Salmonella* infection is explained by favoring an innate immune system activated state allowing the host to remove or kill the bacteria more rapidly. TGFB1 maintains innate immune factor expression within a range that allows inhibition of bacterial growth during infection, a situation that is beneficial to host *Salmonella* resistance.

Measurement of the transcriptomic response in a limited number of individuals challenged with *Salmonella* identified substantially different responses of major canonical immune pathways. Our gene expression data were quite extensive in the number of responsive genes. This experiment also provided significant new information for many poorly annotated transcripts in the porcine genome, as correlation of their expression pattern to the known regulatory pathways described above may be a clue to their regulation and function in porcine *Salmonella* infection. Combining these results, we hypothesize that much of the gene expression response in the peripheral blood of pigs after *Salmonella* challenge could be directly due to higher levels of IFN-γ, TNF, and TGFB1 in the bloodstream. Intriguingly, two regulators, TGFB1 and TRP53, were found to regulate a large number of genes in peripheral blood, and could also regulate intracellular *Salmonella* growth in macrophages. However, the detailed mechanisms underlying this observation remain to be discovered.

## Additional files

**Additional file 1.** **GEREA.** Gene expression regulator enrichment analysis.

**Additional file 2.** **Gene expression data**. The complete gene expression data.

**Additional file 3.** **Differentially expressed transcripts.** The full list of differentially expressed transcripts.

**Additional file 4.** **Over-represented pathways.** The significantly over-represented pathways in cluster A, C, D, E, and F.

**Additional file 5.** **Regulators of differentially expressed genes.** Full list of regulators of differentially expressed genes.

**Additional file 6.** **Real-time PCR primers.**

**Additional file 7.** **Real-time PCR result.** Raw data and statistical results for real-time PCR.

**Additional file 8.** ***Salmonella*** **intracellular salmonella growth assay.** Details of the *Salmonella* intracellular salmonella growth assay.
