# Supplementary material for: Regulators of Salmonella-host interaction identified by peripheral blood transcriptome profiling: roles of TGFB1 and TRP53 in intracellular Salmonella replication in pigs
Source: Vet Res. 2018 Dec 12;49:121. doi: 10.1186/s13567-018-0616-9 (PMC6292071; doi:10.1186/s13567-018-0616-9)
Supplement: Supplementary file 1 — Additional file 1. GEREA. Gene expression regulator enrichment analysis. [file 13567_2018_616_MOESM1_ESM.docx]

**Gene Expression Regulator Enrichment Analysis**

1. **Construction the gene expression regulation database**

Abstract retrieval is relied on NCBI Entrez Programming Utilities search engine (E-utilities, Esearch and Efetch) to filter related articles. However, results from NCBI search engine had noise and significantly decreased text-mining performance. Each abstract was analyzed using a SVM classifier built on word features extracted from known examples. The abstracts that passed the SVM classifier were manually curated by biologists to extract relationships between gene expression and regulation.

We used simplified gene expression regulator–target Links to represent the relationship between gene expression and regulation. The standard definition of “gene expression regulator” that we provide in the Links is– a gene that can change the expression of a target gene. The Links include three major functional elements– the gene expression regulator (regulon), the target gene, and the direct link connecting the regulon and the target.

To date, the curation team has reviewed more than 53 229 publications and has curated 39 939 gene expression regulation interactions, richly annotating them in terms of the evidence they provide and the context in which they occur in the abstracts. The importance of manual curation is clear, as we were often able to extract more Links for a given gene or protein than the number of Links identified automatically by text mining tools. Furthermore, the detailed manual curation permitted us to richly annotate these Links and to place them in their relevant context. This contextual annotation includes details of the supporting publication, genes studied, the species, the effect of regulation, and several other fields.

A flexible web-based interface allows simple searching of GEREDB. This interface has been developed in close collaboration with our biologists to ensure that the interface is easy to use for the end user. Moreover, the flat file format of the rational database in the back end can be downloaded and analyzed locally by the user. On the GREDB search page, one has an option of searching the Links for “Regulator” or “Target”. As the data in GREDB are organized according to genes, Links search allows one to retrieve data of interest using NCBI official gene symbol (gene name). Genes are frequently known by several different names or symbols, which are called synonyms. We map these synonyms of the gene stored in GREDB to Entrez Gene to avoid any ambiguous search terms. From the Links search results page, information related to the Links of interest can be obtained, including official gene symbol and gene id of the regulators and targets, and the effect of the regulator on the target in each Link. The users can also use a program to access the search the result page by passing URL parameters, the “gene_symble” and “search_for”. From the Evidence page, details of the evidences supporting the Link, including the original abstract and the sentence supporting the Link (highlighted in red), can be obtained.

1. **Development of the GEREA software**

Building on this data, the GEREA bioinformatics tool was used to facilitate systems-level investigations of the gene expression regulations. Here, we define active regulator as a gene that has targets that occur in lists of differentially expressed genes with significantly higher frequencies than expected. After the regulator–target regulation Links were built, the gene expression profiling data was loaded on the targets of the regulators. An expected ratio in the background (P0) and an observed ratio (P1) in the list of differentially expressed genes were calculated. If P1 is significantly higher than P0, then we considered the targets of the regulator to be significantly enriched in the differentially expressed genes, indicating that the targets were regulated by the regulator. Statistical analysis was performed based on cumulative hypergeometric distribution and corrected by Benjamini and Hochberg method (FDR).

Suppose that n genes have been measured in the transcription profiling experiment, k genes were differentially expressed. For the n genes, s genes are targets of the regulated through transcription factors (hereafter this text will be abbreviated as targets) in the network. For the k differentially expressed genes, x genes are targets of the regulator in the network. An expectation ratio P0 = s/n and an observed ratio P1 = x/k was calculated. If P1 is significantly higher than P0, then we say the targets of the microRNA were significantly enriched in the differentially expressed genes, indicating that those genes were regulated or controlled by the regulator. Statistical analysis is performed based on cumulative hypergeometric distribution (1).

$$P=f\left( k,x,n,s \right)=\frac{\binom{s}{x}\binom{n-s}{k-x}}{\binom{n}{k}}=\frac{s!\left( n-s \right)!k!\left( n-k \right)!}{x!\left( s-x \right)!\left( k-x \right)!\left( (n-s)-(k-x) \right)!n!} (1)$$

Utilizing the networks, the software GEREA has been developed to analyze the function of regulators using gene expression profiling data as input. There are three major steps built into the program. 1) Build the regulation network based on the links in the network file. 2) Load the transcriptome data in the data file. The program matches each gene id in the transcriptome to the target gene ids in the network, which both are NCBI refseq accession numbers. If they are found, then the expression data is mapped to the target gene, otherwise, the data is omitted. 3) Perform a statistical analysis of the network. The values of *n*, *k*, *s*, and *x* were counted. Statistical analysis was performed using the Fisher’s exact test, which was calculated using the BigNum (GMP library). The statistical result and the network were then printed to files. The statistical output file was formatted as a tab-delimitated text file. The first line consists of description, and the following lines describe the microRNA name, values of *n*, *s*, *k*, and *x* as well as the P values and FDR. The network output file contains the links for each network that has been analyzed. Several utility programs have also been developed, such as a program to export the network to Cytoscape for advanced network graphing. The GEREA tool is available at GEREDB website.
