# Supplementary material for: Regulators of Salmonella-host interaction identified by peripheral blood transcriptome profiling: roles of TGFB1 and TRP53 in intracellular Salmonella replication in pigs
Source: Vet Res. 2018 Dec 12;49:121. doi: 10.1186/s13567-018-0616-9 (PMC6292071; doi:10.1186/s13567-018-0616-9)
Supplement: Supplementary file 4 — Additional file 4. Over-represented pathways. The significantly over-represented pathways in cluster A, C, D, E, and F. [file 13567_2018_616_MOESM4_ESM.docx]

**The significantly over represented pathway in cluster A, C, D, E, and F**

| Cluster name | Pathway name | Pathway source | Pathway Id | Gene count in pathway | Gene count in cluster | Ratio | Corrected *p*-value |
| --- | --- | --- | --- | --- | --- | --- | --- |
| Cluster A | Metabolism | REACTOME | 17674 | 1414 | 55 | 0.038897 | 0.00992 |
| Cluster A | Immune System | REACTOME | 17702 | 884 | 50 | 0.056561 | 1.03E-05 |
| Cluster A | Disease | REACTOME | 16877 | 879 | 42 | 0.047782 | 0.001944 |
| Cluster A | Innate Immune System | REACTOME | 16733 | 450 | 27 | 0.06 | 0.001715 |
| Cluster A | Adaptive Immune System | REACTOME | 19062 | 484 | 23 | 0.047521 | 0.032108 |
| Cluster A | Phagosome | KEGG | 10382 | 165 | 16 | 0.09697 | 3.54E-04 |
| Cluster A | Glycogen storage diseases | REACTOME | 18944 | 228 | 16 | 0.070175 | 0.006381 |
| Cluster A | Lysosome | KEGG | 4357 | 123 | 15 | 0.121951 | 4.84E-05 |
| Cluster A | Class I MHC mediated antigen processing & presentation | REACTOME | 16844 | 203 | 13 | 0.064039 | 0.025319 |
| Cluster A | Platelet activation, signaling and aggregation | REACTOME | 17716 | 186 | 12 | 0.064516 | 0.033589 |
| Cluster A | Cytokine Signaling in Immune system | REACTOME | 17370 | 194 | 12 | 0.061856 | 0.04391 |
| Cluster A | Toll-Like Receptors Cascades | REACTOME | 18742 | 127 | 11 | 0.086614 | 0.009869 |
| Cluster A | Toll Like Receptor 4 (TLR4) Cascade | REACTOME | 19911 | 109 | 10 | 0.091743 | 0.010378 |
| Cluster A | Latent infection of Homo sapiens with *Mycobacterium tuberculosis* | REACTOME | 17474 | 32 | 9 | 0.28125 | 9.50E-06 |
| Cluster A | Phagosomal maturation (early endosomal stage) | REACTOME | 17472 | 32 | 9 | 0.28125 | 9.50E-06 |
| Cluster A | MyD88:Mal cascade initiated on plasma membrane | REACTOME | 19563 | 81 | 9 | 0.111111 | 0.007613 |
| Cluster A | Toll Like Receptor 2 (TLR2) Cascade | REACTOME | 16707 | 81 | 9 | 0.111111 | 0.007613 |
| Cluster A | Activated TLR4 signalling | REACTOME | 19167 | 99 | 9 | 0.090909 | 0.013254 |
| Cluster A | Osteoclast differentiation | KEGG | 10372 | 126 | 9 | 0.071429 | 0.049716 |
| Cluster A | Antigen processing-Cross presentation | REACTOME | 18791 | 76 | 8 | 0.105263 | 0.011199 |
| Cluster A | Signaling by Interleukins | REACTOME | 18761 | 100 | 8 | 0.08 | 0.043623 |
| Cluster A | Glucose metabolism | REACTOME | 17651 | 65 | 7 | 0.107692 | 0.018109 |
| Cluster A | Fc epsilon RI signaling pathway | KEGG | 708 | 69 | 7 | 0.101449 | 0.024276 |
| Cluster A | Glycerophospholipid biosynthesis | REACTOME | 19211 | 77 | 7 | 0.090909 | 0.039861 |
| Cluster A | Iron uptake and transport | REACTOME | 17954 | 40 | 6 | 0.15 | 0.009197 |
| Cluster A | Toll-like receptor signaling pathway | INOH | 16122 | 40 | 6 | 0.15 | 0.009197 |
| Cluster A | Insulin receptor recycling | REACTOME | 19277 | 25 | 5 | 0.2 | 0.009385 |
| Cluster A | Transferrin endocytosis and recycling | REACTOME | 17199 | 26 | 5 | 0.192308 | 0.009759 |
|  |  |  |  |  |  |  |  |
| Cluster C | Metabolism | REACTOME | 17674 | 1414 | 7 | 0.00495 | 0.029614 |
| Cluster C | Disease | REACTOME | 16877 | 879 | 5 | 0.005688 | 0.032846 |
| Cluster D | Cell Cycle | REACTOME | 17635 | 501 | 9 | 0.017964 | 3.19E-04 |
| Cluster D | Cell Cycle, Mitotic | REACTOME | 16819 | 423 | 8 | 0.018913 | 5.38E-04 |
| Cluster D | Mitotic G1-G1/S phases | REACTOME | 18886 | 124 | 6 | 0.048387 | 1.05E-04 |
| Cluster E | Metabolism of proteins | REACTOME | 17734 | 555 | 13 | 0.023423 | 5.64E-04 |
| Cluster E | Gene Expression | REACTOME | 19038 | 723 | 13 | 0.017981 | 0.006168 |
| Cluster E | Translation | REACTOME | 17506 | 138 | 9 | 0.065217 | 2.09E-05 |
| Cluster E | Eukaryotic Translation Elongation | REACTOME | 18743 | 83 | 8 | 0.096386 | 9.92E-06 |
| Cluster E | GTP hydrolysis and joining of the 60S ribosomal subunit | REACTOME | 19753 | 102 | 8 | 0.078431 | 1.25E-05 |
| Cluster E | Cap-dependent Translation Initiation | REACTOME | 18051 | 108 | 8 | 0.074074 | 1.30E-05 |
| Cluster E | Eukaryotic Translation Initiation | REACTOME | 18327 | 108 | 8 | 0.074074 | 1.30E-05 |
| Cluster E | L13a-mediated translational silencing of Ceruloplasmin expression | REACTOME | 19353 | 101 | 8 | 0.079208 | 1.55E-05 |
| Cluster E | Ribosome | KEGG | 756 | 142 | 8 | 0.056338 | 4.80E-05 |
| Cluster E | Eukaryotic Translation Termination | REACTOME | 17591 | 80 | 7 | 0.0875 | 1.73E-05 |
| Cluster E | Peptide chain elongation | REACTOME | 16999 | 80 | 7 | 0.0875 | 1.73E-05 |
| Cluster E | Nonsense Mediated Decay (NMD) independent of the Exon Junction Complex (EJC) | REACTOME | 17898 | 85 | 7 | 0.082353 | 2.33E-05 |
| Cluster E | Formation of a pool of free 40S subunits | REACTOME | 18890 | 91 | 7 | 0.076923 | 3.34E-05 |
| Cluster E | SRP-dependent cotranslational protein targeting to membrane | REACTOME | 18716 | 101 | 7 | 0.069307 | 4.82E-05 |
| Cluster E | Nonsense Mediated Decay (NMD) enhanced by the Exon Junction Complex (EJC) | REACTOME | 17890 | 99 | 7 | 0.070707 | 4.91E-05 |
| Cluster E | Nonsense-Mediated Decay (NMD) | REACTOME | 17718 | 99 | 7 | 0.070707 | 4.91E-05 |
| Cluster F | Gene Expression | REACTOME | 19038 | 723 | 26 | 0.035961 | 3.17E-04 |
| Cluster F | Metabolism of proteins | REACTOME | 17734 | 555 | 21 | 0.037838 | 0.001088 |
| Cluster F | Translation | REACTOME | 17506 | 138 | 15 | 0.108696 | 1.47E-07 |
| Cluster F | Ribosome | KEGG | 756 | 142 | 14 | 0.098592 | 4.82E-07 |
| Cluster F | Eukaryotic Translation Elongation | REACTOME | 18743 | 83 | 13 | 0.156627 | 5.89E-08 |
| Cluster F | Formation of a pool of free 40S subunits | REACTOME | 18890 | 91 | 13 | 0.142857 | 9.67E-08 |
| Cluster F | GTP hydrolysis and joining of the 60S ribosomal subunit | REACTOME | 19753 | 102 | 13 | 0.127451 | 1.03E-07 |
| Cluster F | L13a-mediated translational silencing of Ceruloplasmin expression | REACTOME | 19353 | 101 | 13 | 0.128713 | 1.04E-07 |
| Cluster F | SRP-dependent cotranslational protein targeting to membrane | REACTOME | 18716 | 101 | 13 | 0.128713 | 1.04E-07 |
| Cluster F | Cap-dependent Translation Initiation | REACTOME | 18051 | 108 | 13 | 0.12037 | 1.53E-07 |
| Cluster F | Eukaryotic Translation Initiation | REACTOME | 18327 | 108 | 13 | 0.12037 | 1.53E-07 |
| Cluster F | Eukaryotic Translation Termination | REACTOME | 17591 | 80 | 12 | 0.15 | 1.03E-07 |
| Cluster F | Peptide chain elongation | REACTOME | 16999 | 80 | 12 | 0.15 | 1.03E-07 |
| Cluster F | Nonsense Mediated Decay (NMD) independent of the Exon Junction Complex (EJC) | REACTOME | 17898 | 85 | 12 | 0.141176 | 1.17E-07 |
| Cluster F | Nonsense Mediated Decay (NMD) enhanced by the Exon Junction Complex (EJC) | REACTOME | 17890 | 99 | 12 | 0.121212 | 4.46E-07 |
| Cluster F | Nonsense-Mediated Decay (NMD) | REACTOME | 17718 | 99 | 12 | 0.121212 | 4.46E-07 |
