# Supplementary material for: Regulators of Salmonella-host interaction identified by peripheral blood transcriptome profiling: roles of TGFB1 and TRP53 in intracellular Salmonella replication in pigs
Source: Vet Res. 2018 Dec 12;49:121. doi: 10.1186/s13567-018-0616-9 (PMC6292071; doi:10.1186/s13567-018-0616-9)
Supplement: Supplementary file 6 — Additional file 6. Real-time PCR primers. [file 13567_2018_616_MOESM6_ESM.docx]

**Real-time PCR primers**

| Gene Symbol | Acession NO. | Forward primer | Revise primer |
| --- | --- | --- | --- |
| Abca1 | NM_001317080 | GAGGCGCGGCTGAAGGAGACTA | CCAAACCCAAAAGCCACAGGAGA |
| Actb | XM_003124280 | GGACGAGGCCCAGAGCAAGAGA | CCCGGCCAGCCAGGTCCAG |
| Alox5ap | NM_001164001 | TGGCCATCGTCACCCTCATCAG | ACCCGGAATTCAGCAGCCAGTTAG |
| Basp1 | XM_003483806 | CAGCAGCCACGGAAGCACCCAGTT | CACCCCCTCCCTCCCTCCCTTGAT |
| Bcl2l11 | XM_021085830 | GGAAGGGGCCAAAGGTCT | AAGGGGGCAAAATATTCTGTGTTA |
| Bnip3l | XM_001927592 | GAATAAAAGCACCGTGACAACCAG | GGGCCACCAAATTTACTTCCTTC |
| Bub1b | XM_021097818 | CTCATTTCGCATCCACTCCTTTTC | GGGCCATCTGCAGTCTCATTAGTG |
| Casp1 | NM_214162 | ACACGCCTTGCCCTCATAATCTG | ATACGACCCCTTGCTTCTCACCAC |
| Casp4 | XM_003129812 | CGCAACTGCCTCGGTCTGA | ATCTGGGCTTTAACGCTTGGTGT |
| Ccl5 | NM_001129946 | TCCATGGCAGCAGTCGTCTTTATC | AGCCCCTTCTCCCTCTCCTCTTC |
| Ccr1 | NM_001001621 | CCGGCGGCGCTTCTACCTG | AGCTGCCACATCCCTCAACTTTCA |
| Cd14 | NM_001097445 | CGCCAGCGCCAACCCGAAGCAGTA | CCAGCCCGGGATTGTCAGATAGGT |
| Cd163 | NM_213976 | GCTCTGGGCGCATCACTCTGTTC | CCAAATGGGCCCTGTTCCTTCC |
| Cebpb | NM_001199889 | CGACGACTACGGGGGCAAGAACTG | CGGGCGGGCTGGACGACGAGGATG |
| Cks1b | NM_001243542 | TGCCCAAGAAGCCAAAGAAATGAA | ACAACACCTGGCGGGGAAAAA |
| Cyba | NM_214267 | CATTGCGGCGGGCGTGTTGGT | GTTGCTGGGCGGCTGCTTGATGGT |
| Dut | XM_003353370 | GCGCCGCGGGATATGACCTGTA | TTCTGTTCTTGGCGTAAAT |
| Edn1 | NM_213882 | CCCCGGGCCCGACTCTGC | AGGCGCCTGCGTGGGACAAC |
| Gadd45b | XM_005654701 | CCGGCCTCTAATTCCCCCACTGTC | GGCGCTCACCGTCTGCATCTTTTG |
| Gapdh | NM_001206359 | AGGCCGGGGCTCACTTG | CTCCAGGCGGCAGGTCAGAT |
| Hagh | XM_021086829 | CGCTGCCTGCTCTGACCGACAAT | TTCCCGCAGCCAGCCACAAACAAG |
| Ifrd2 | XM_001925104 | TTGGCCCTGGCAGCTCACCTACTT | GGCGCCACCCACACCACAG |
| Lgals1 | NM_001001867 | TCTCCTCAGTCATGGCTTGTGGTC | TCTGGCAGCTTGATGGTGAGGTCT |
| Ptpa | XM_001926077 | GTGTGGGGCCTGGACGACTT | GGGGGCGGGCATAGGAAC |
| Rpsa | NM_001037146 | TTGCTGGCCGCTTCACTCCTG | CTGCGGCCTGCTCTTCCTTTTC |
| Srgn | XM_013990411 | TCTTTCTGGGGTTTGATGTGGAT | GGAGGTAGGATTCTGTTGGATTCA |
| Tgfb2 | XM_021064295 | TGCTGCCTGCGTCCACTTTACATT | ACTGCCGTCGCCATCATCAACATC |
| Tgfbi | XM_021084784 | GGATTGACGCCCGGACGAAGAA | AGCCCCGATGCCCCCACTCAC |
| Unc5b | XM_005671044 | CCTCGCCCCTCCCGCCAGAAGT | CACGCGCAAGCACACCCCAGAGAT |
| Ybx1 | XM_021096922 | CGCAGCCGGCCCCAGTCAC | GCCTCCGCACCCTTTTCTCCTTCA |
