# Supplementary material for: Regulators of Salmonella-host interaction identified by peripheral blood transcriptome profiling: roles of TGFB1 and TRP53 in intracellular Salmonella replication in pigs
Source: Vet Res. 2018 Dec 12;49:121. doi: 10.1186/s13567-018-0616-9 (PMC6292071; doi:10.1186/s13567-018-0616-9)
Supplement: Supplementary file 8 — Additional file 8. Salmonella intracellular salmonella growth assay. Details of the Salmonella intracellular salmonella growth assay. [file 13567_2018_616_MOESM8_ESM.docx]

**Salmonella intracellular salmonella growth assay**

***Bacterial strain, cells, and culture media***

*Salmonella typhimurium* strain LT2 (ATCC700720) is virulent to humans and animals. Luria broth and M9 minimal medium were used to culture Salmonella bacteria. Carbon sources such as glucose, glycerol, citrate, or acetate were added to the M9 minimal medium to a final concentration of 0.2%. To establish calcium- or magnesium-limited conditions, CaCl_2_ or MgSO_4_ was omitted from the M9 minimal medium. Mouse macrophages (RAW 264.7) were cultured in Dulbecco’s minimal essential medium containing 10% fetal bovine serum (FBS, DMEM). Proteose-peptone-elicited macrophages were harvested 3 days after elicitation with 1.5 mL of 10% proteose peptone.

The bone marrow-derived macrophages were isolated using Ronan’s method. The frozen cells were thawed rapidly in a water bath at 37 °C and then slowly diluted by dropwise addition of complete medium over 2-3 min to avoid shock from the sudden dilution of DMSO. After washing to remove the DMSO, the cells were cultured in RPMI 1640 complete medium (Sigma-Aldrich), 10% heat-inactivated FBS (Gibco), penicillin-streptomycin (Invitrogen), and GlutaMAX-I supplement (Invitrogen). Bone-marrow-derived macrophages were obtained by culturing bone marrow cells for 5-7 days in the presence of CSF-1 (0.5 ng/mL), essentially as described previously for mouse macrophages. Invasion and intracellular replication assays were set up by seeding 1 × 10^5^ cells into each well of a 24-well microtiter dish and incubating overnight.

***Invasion and intracellular replication assays***

A 1-mL standing-overnight culture was prepared by inoculating L-broth with Salmonella bacteria from frozen glycerol stocks and incubating at 37 °C. Two hours prior to infection, Salmonella was diluted to 1:20 in fresh L-broth and incubated until reaching the mid-logarithmic growth phase. Bacterial cells were pelleted and washed in PBS before adding them to each microtiter well. When the target genes were tested, they were added to the cells 24 h prior to bacterial addition. The bacteria inoculation was carried out using 5 μL of Salmonella culture, and the microtiter plates were centrifuged (162 × *g*, 10 min, 23 °C), followed by 2 h of incubation at 37 °C in a 5% CO_2_ atmosphere. Monolayers were washed 3 times with PBS and then incubated for another 2 h in fresh media containing 100 μg/mL of gentamicin. This treatment kills extracellular bacteria but does not affect the viability of intracellular organisms. Monolayers were washed three times with PBS, and 0.2 mL of a 1% Triton X-100 solution was added. This was followed by a 5-min incubation period to release intracellular bacteria. L-broth (0.8 mL) was added, appropriate dilutions were spread onto L-agar plates, and colony-forming units (cfu) were counted. The experiment was repeated three times.

***Statistical analysis***

Results are shown as means ± standard deviation (S.D.) of at independently duplicated experiments. Data were analyzed using one-way ANOVA followed by a multiple comparison test (Tukey honest significant differences) to determine which conditions were significantly different from each other. All statistical analyses were performed using SAS software. Probability values ≤ 0.05 and ≤ 0.01 were taken to indicate statistical significance.

***Macrophages infection by Salmonella bacterial (40×)***


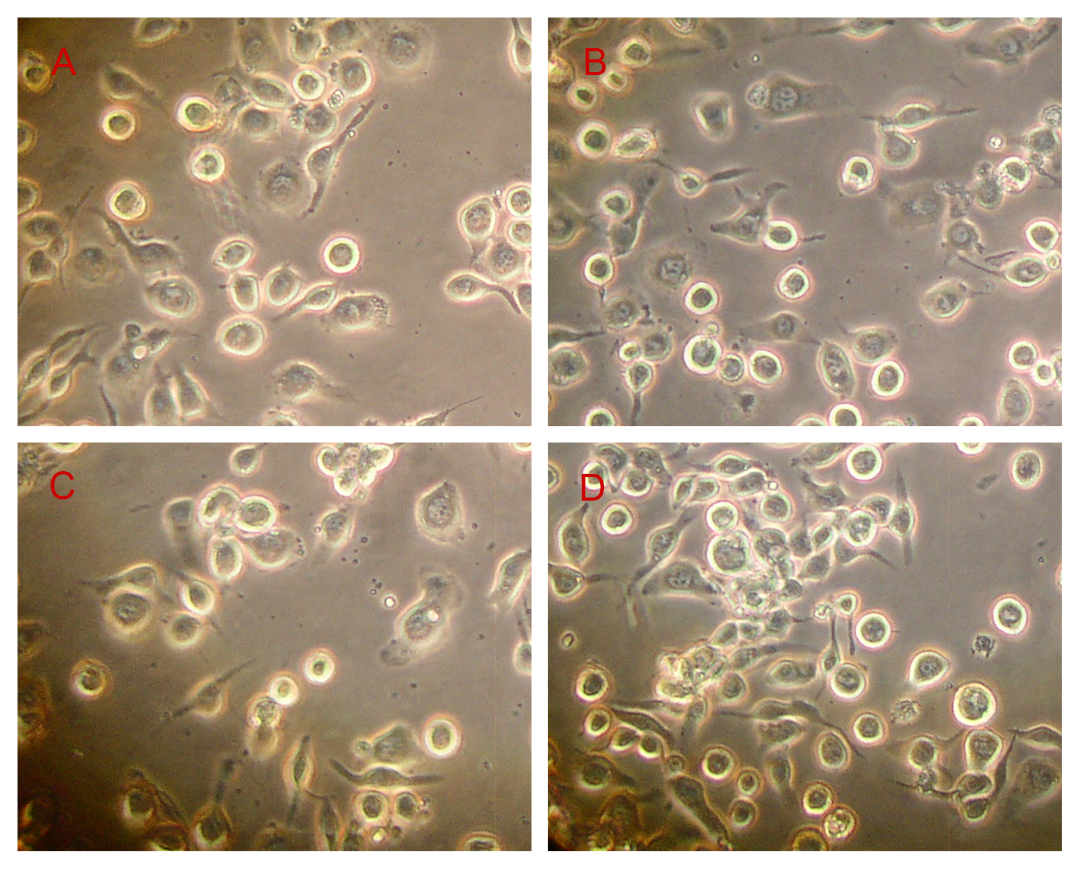


A: Bone marrow-derived macrophages (before infection); B: Bone marrow-derived macrophages infection by Salmonella bacteria (24 h post-infection); C: Mouse macrophage (Raw 264.7, before infection); D: Mouse macrophage (Raw 264.7) infected with Salmonella bacteria (24 h post-infection).
